# Study on the emergency capacity of coal mine enterprises in Longdong Area based on D-FAHP method

**DOI:** 10.1038/s41598-023-34618-6

**Published:** 2023-05-09

**Authors:** Fengfeng Yang, Jufeng Zhang, Bangxin Jin, Siyang Wang, Caixia Xi

**Affiliations:** 1 College of New Energy, Long Dong University, 745000 Qingyang, China; 2 State Key Laboratory of Fire Science, University of Science and Technology of China, Hefei, 230026 China

**Keywords:** Risk factors, Engineering, Energy infrastructure

## Abstract

Emergency capability assessment is a complex system with multiple factors, variables and levels. Incomplete and uncertain assess information often occurs during assessment. Based on this, a method combining D-number theory and fuzzy analytic Hierarchy process (FAHP) is proposed to study the emergency capacity of coal enterprises in Longdong area. On the basis of analyzing the limitation of D-S evidence theory, the D-number theory was optimized and improved. According to the principles of systematicness, feasibility, scientificity and timeliness, a hierarchical structure model of enterprise emergency capability assessment was constructed from the perspective of pre-incident, mid-incident and post-incident, which consisted of 4 first-level indicators and 18 s-level indicators. The weight and importance of the assessment index of emergency response capability are calculated by organically integrating the D-number preference relation with the hierarchy structure. Combined with the assessment results of experts, a quantitative analysis and evaluation of the emergency response capacity of a coal enterprise was conducted by using FAHP. The comprehensive score of the enterprise's emergency response capability was 80.45, and the level of emergency response capacity was "good". The research results show that the D-FAHP method has high reliability in evaluating the emergency response capability of coal enterprises, avoiding the impact of uncertain and incomplete information on the assessment results. This can not only effectively identify the weak links in emergency management, but also meet the emergency decision-making needs of enterprises in the emergency state, which has important guiding significance to improve the ability and level of enterprise emergency management.

In 2021, China's energy consumption totaled 5.24 billion tons of standard coal, accounting for 56% of the total energy consumption. The composition of coal—based energy resources will not change in the short term. Longdong region is located in Pingliang and Qingyang cities in eastern Gansu Province with rich coal resources, in the southwestern part of the Ordos Basin, where Gansu, Ningxia and Shaanxi provinces meet. In 2014, the National Energy Administration approved *The Development Plan of Longdong Energy Base*. At present, Longdong is building a domestic leading and world-class modern comprehensive energy base. The 14th Five-Year Plan of Gansu Province also clearly points out that it is necessary to speed up the green development and utilization of Longdong coal and the construction of conversion base, serve to ensure the national energy security. Coal fields in Gansu Province are mainly distributed in the middle and east. Pingliang coal geological reserves of 65 billion ton; Qingyang area of the predicted reserves of 236 billion ton; Longdong coal resources accounted for more than 94% of Gansu province^1–3^. At present, the implementation of carbon neutrality and carbon peak action will promote the continuous self-revolution of coal mining technology. Sudden events with new characteristics may occur in coal enterprises. Based on this situation, in view of the possible emergencies in the development process of coal resources in Longdong region, qualitative and quantitative research on its emergency capacity is carried out to improve the emergency management ability of Longdong energy base, which is of great practical significance to ensure coal safety and green mining.

It’s well known that coal is a traditional labor-intensive industry with more than five million employees. Coal mining is a high-risk industry, and the working conditions of coal miners are relatively dangerous^4–6^. According to statistics, China accounts for 70% of the world's coal mine casualties^7^. At present, the major accident disasters still occur frequently in Chinese coal mine, and the weak emergency ability is the main reason that leads to the serious consequences^8，9^. However, when the enterprise has a sound and perfect emergency management system, the loss caused by accidents will be reduced to 7%^10^. It can be seen that emergency capacity building play a vital role in coal enterprise, and the key to strengthen emergency capacity building is to carry out dynamic and continuous scientific assessment^11，12^.

In foreign countries, the earliest research on emergency management capability mainly focuses on the political field, and gradually extends to the evaluation of emergency management capability for typhoon, tsunami, hurricane, flash flood, waterlogging, fire and other emergencies^13,14^. In recent years, there are relatively few foreign studies on the emergency management capacity in the field of coal mine. However, the successful experience of emergency management in the disaster field has reference value for the study of the emergency capacity of coal mining enterprises.

In China, Miao Chenglin^15^ proposed a theoretical model of the influence of habit field on the emergency response capability of coal mine emergencies, and verified the positive effect of this model on the emergency response capability with AMOS software. Sheng Yong^12,16^ combed and constructed an enterprise emergency capability assessment system based on the bottom-line thinking and the method of scenario construction and deduction. Based on extension theory, some scholars studied the factors affecting emergency response ability from different perspectives and constructed an assessment system^17,18^. Some scholars^19,20^ have integrated the open class method, entropy method and support vector machine to build an evaluation system of coal mine emergency capacity. Some scholars have constructed an indicator system of enterprise emergency response capability from the perspective of dynamic capability development and capability maturity, and adopted catastrophe progression to evaluate the emergency response capability^21,22^. In addition, there are structural equation model^23^, fuzzy comprehensive evaluation method^24^, analytic hierarchy process^25,26^ and other evaluation methods.

Zhang^27^ calculated the weight of each index of the evaluation model by using the analytic hierarchy process, and evaluated the miners’ emergency capacity by using the fuzzy comprehensive evaluation method. Chen^28^ proposed a coal mine emergency rescue ability evaluation model, which used intuitionistic fuzzy entropy method to calculate the index weight, and intuitionistic fuzzy weighted average operator to calculate the comprehensive evaluation matrix. According to the characteristics of diversity and uncertainty of information, Qi^29^ proposed a multi-criteria comprehensive evaluation method based on interval binary representation model to avoid information distortion and loss in the process of linguistic information processing. Zhang^30^ believed that the emergency assessment information was incomplete and uncertain, and proposed the method of using evidence theory to evaluate the city's emergency response capacity. Yang^31^ analyzed the development and evolution process of coal mine emergency response capability by using Logistic curve, and evaluated the emergency response capability of coal mine enterprises based on fuzzy analytic hierarchy process.

In conclusion, in the assessment system of coal enterprises' emergency response capacity, some indicators are too qualitative, and some indicators are not operable or difficult to measure. The indicators are interrelated and influence each other. Meanwhile, the data processing is sketchy. As a result, the assessment results are lack of scientificity. Therefore, it is particularly significant to establish a scientific, measurable and operable index system for the assessment of coal enterprise emergency capacity.

It was difficult for experts to determine the degree of assessment when they used AHP, IAHP, FAHP and other methods to assess. These methods also did not consider the effect of incomplete information, which affected the reliability of the evaluation results. In addition, in order to make the evaluation results as independent as possible from personal preferences, the judgment matrix can be constructed through the expert group. But a feature vector that satisfies all judgment matrices is hard to find. In summary, these evaluation methods do not reflect people's perception of "uncertain" and "unknown" information. Based on this, the D number theory is integrated with AHP to avoid the non-consistent phenomenon of judgment matrix. This effectively solves the impact of information incompleteness and uncertainty on the evaluation results and avoids the mutual influence among the evaluation indicators of emergency capability. Furthermore, it can accurately assess the emergency response ability quantitatively, and provide theoretical support for improving the emergency response ability of coal mining enterprises.

## D-S theory

Firstly, the identification framework of D-S theory is defined as the set of N mutually independent elements contained in the research target^32^, which is denoted by $\Theta$.

Suppose m satisfies the mapping $2^{\Theta } \to [0,1][0,1]$. If any subset $A \subseteq \Theta$ and $m\left( \varphi \right) = 0,\sum\limits_{A \subseteq \Theta } {m\left( A \right) = 1}$ are both satisfied, $m\left( A \right)$ is the basic probability function of A, and $2^{\Theta }$ represents the power set of $\Theta$.

Assume that belief function $B_{{B{\text{el}}}}$ satisfies mapping $2^{\Theta } \to [0,1]$. If $B \subseteq A \subseteq \Theta$ and $B_{{B{\text{el}}}} \left( A \right) = \sum {m\left( B \right)}$ are both satisfied, then $B_{{B{\text{el}}}} \left( A \right)$ is called true belief function A.

Let the trust function $P_{Pl}$ satisfy the mapping $2^{\Theta } \to [0,1]$ and $P_{Pl} (A) = 1 - B_{Bel} \left( {\overline{A} } \right)$, then $P_{Pl} \left( A \right)$ is called the likelihood function. The combined D-S synthesis rules are as follows:

1 $$m(A) = \left\{ \begin{gathered} 0,\quad \quad \quad \quad \quad \quad \quad \quad \quad \quad \;A = \varphi \hfill \\ \frac{{\sum\limits_{A \cap B = C} {m_{1} \left( A \right)m_{2} \left( B \right)} }}{1 - K},\quad \;A \ne \varphi \hfill \\ \end{gathered} \right.$$

where $m_{1} \left( A \right)$ and $m_{2} \left( B \right)$ represent the basic probability functions of A and B, K is the conflict factor, and $K = \sum\limits_{A \cap B = \varphi } {m_{1} } \left( A \right)m_{2} \left( B \right)$ represents the degree of conflict between the two evidences.

In practical application, D-S theory requires independent evaluation indexes and complete information, so this limitation makes it more suitable for dealing with uncertain problems with less data. Therefore, Deng^33,34^ proposed an improved D-number theory on the basis of D-S theory.

## D-number theory

### Definition of D-number theory

1. Let there exist A finite nonempty set A and a mapping $D:\Omega \to \left[ {0,1} \right]$ satisfying:
  2 $$\sum\limits_{B \subseteq \Omega }^{{}} {D\left( B \right) \le 1,D\left( \theta \right)} = 0$$
  then the mapping *D* is called D number, where *B* is a subset of $\Omega$, and $\theta$ is empty set. If $\sum\limits_{B \subseteq \Omega }^{{}} {D\left( B \right)} = 1,$ the information represented by D number is complete; If $\sum\limits_{B \subseteq \Omega } {D\left( B \right)} < 1$, the information is incomplete.
2. There exists D-number D and non-empty finite set C, then the information integrity Q of D can be quantified as follows.
  3 $$Q = \sum\limits_{B \subseteq \Omega }^{{}} {D\left( B \right)}$$
3. Let the discrete set $\Omega { = }\left\{ {b_{1} ,b_{2} , \ldots ,b_{i} , \ldots ,b_{n} } \right\}$, where, $b_{i} \in R$, and when $i \ne j$, $b_{i} \ne b_{j}$, then the D number can be expressed as:
  4 $$D\left( {\left\{ {b_{1} } \right\}} \right) = v_{1} ,D\left( {\left\{ {b_{2} } \right\}} \right) = v_{2} , \ldots ,D\left( {\left\{ {b_{n} } \right\}} \right) = v_{n}$$
  or simply expressed as:
  5 $$D = \left\{ {\left( {b_{1} ,v_{1} } \right),\left( {b_{2} ,v_{2} } \right), \ldots ,\left( {b_{n} ,v_{n} } \right)} \right\}$$
  where $$v_{i} \ge 0$$ and $$\sum\limits_{i = 1}^{n} {v_{i} } \le 1$$.
4. (Permutation invariance), if two D number: $D_{1} = \left\{ {\left( {b_{1} ,v_{1} } \right),\left( {b_{2} ,v_{2} } \right), \ldots ,\left( {b_{n} ,v_{n} } \right)} \right\}$ $D_{2} = \left\{ {\left( {b_{n} ,v_{n} } \right), \ldots ,\left( {b_{i} ,v_{i} } \right), \ldots ,\left( {b_{1} ,v_{1} } \right)} \right\}$, Then $D_{1} \Leftrightarrow D_{2}$.
5. Let D number: $D = \left\{ {\left( {b_{1} ,v_{1} } \right), \ldots ,\left( {b_{i} ,v_{i} } \right), \ldots ,\left( {b_{n} ,v_{n} } \right)} \right\}$, then its fusion can be expressed as:
  6 $$I\left( D \right) = \sum\limits_{i = 1}^{n} {b_{i} v_{i} }$$

The $I$ value is the fusion of D numbers. For example, an evaluation result can be expressed as:$D = \left\{ {\left( {0.5,1.0} \right)} \right\}$, in the form of D-number. After fusion, it can be obtained as:$I\left( D \right) = 0.5 \times 1.0 = 0.5$

### D-number preference relation

The fuzzy preference relation reflects the relative importance of the evaluator to the decision-making scheme to a certain extent (represented by "$\succ$")^35^. Therefore, the fuzzy preference relation is adopted to construct the decision-making matrix.

Let there exist evaluation sample set $A = \left\{ {A_{1} ,A_{2} , \ldots ,A_{n} } \right\}$, whose fuzzy preference relation is $\mu_{R} :A \times A \to [0,1]$ expressed as $R = [r_{ij} ]_{n \times n}$ in matrix form.

7 $$\begin{gathered} \;\;\;\;\;\;\;\;\;\;\;\;\;\;\;\;\;\;\;\;\;A_{1} \;\;A_{2} \;\;\; \cdots \;\;\;A_{n} \hfill \\ R = \begin{array}{*{20}c} {A_{1} } \\ {A_{2} } \\ \vdots \\ {A_{n} } \\ \end{array} \;\;\;\;\left[ \begin{gathered} r_{11} \;\;\;r_{12} \;\;\; \cdots \;\;\;r_{1n} \hfill \\ r_{21} \;\;\;r_{22} \;\;\; \cdots \;\;\;r_{2n} \hfill \\ \;\; \vdots \;\;\;\;\; \vdots \;\;\;\;\; \ddots \;\;\;\; \vdots \hfill \\ r_{n1} \;\;\;r_{n2} \;\;\; \cdots \;\;\;r_{nn} \hfill \\ \end{gathered} \right] \hfill \\ \end{gathered}$$

where $$r_{ij} \ge 0;$$ $$r_{ij} + r_{ij} = 1,\forall i,j \in \left\{ {1,2, \ldots ,n} \right\};$$ $$r_{ii} = 0.5,\forall i \in \left\{ {1,2, \ldots ,n} \right\}$$.

The meaning of matrix element *r*_*ij*_ is the relative importance of $A_{i}$ and $A_{j}$, which is expressed as:

$$r_{ij} = \mu_{R} \left( {A_{i} ,A_{j} } \right) = \left\{ \begin{gathered} 0,\quad \quad \quad \quad A_{j} {\text{ is obviously more important than }}A_{i} \hfill \\ \in \left( {{0},{0}{\text{.5}}} \right),\;A_{j} {\text{ is more important than }}A_{i} {\text{ to some extent}} \hfill \\ {0}{\text{.5}},\quad \quad \quad \,A_{i} {\text{ is indistinguishable from }}A_{j} \hfill \\ \in \left( {{0}{\text{.5}},1} \right),\;A_{i} {\text{ is more important than }}A_{j} {\text{ to some extent}} \hfill \\ 1,\quad \quad \quad \quad A_{i} {\text{ is obviously more important than }}A_{j} \hfill \\ \end{gathered} \right.$$

For example, if 10 experts are selected to evaluate the two schemes A1 and A2, the following results may occur:

1. Eight people thought A_1_ $\succ$ A_2_ with an importance of 0.7, and the other two people thought A_1_ $\succ$ A_2_ with an importance of 0.6.
2. Six people thought A_1_ $\succ$ A_2_ with an importance of 0.8, while the other four people did not evaluate the importance of A_1_ and A_2_ for prudent consideration or other reasons.

Due to the limitation of fuzzy preference relation, a reasonable matrix cannot be constructed when the information is uncertain. Therefore, D mathematical theory is adopted to improve the fuzzy preference relation, so that it can be applied in the field of uncertain and incomplete information. The improved evaluation matrix can be simply referred to as D-number preference matrix^36^.

Assume a set of assessment data $A = \left\{ {A_{1} ,A_{2} , \ldots ,A_{n} } \right\}$, and its D-number preference relationship is:$R_{D} :A \times A \to D$.expressed as $R_{D} = [D_{ij} ]_{n \times n}$ in matrix form:

8 $$\begin{gathered} \;\;\;\;\;\;\;\;\;\;\;\;\;\;\;\;\;\;\;\;\;\;\;\;A_{1} \;\;\;A_{2} \;\;\; \cdots \;\;\;A_{n} \hfill \\ R_{D} = \begin{array}{*{20}c} {A_{1} } \\ {A_{2} } \\ \vdots \\ {A_{n} } \\ \end{array} \;\;\;\left[ \begin{gathered} D_{11} \;\;\;D_{12} \;\;\; \cdots \;\;\;D_{1n} \hfill \\ D_{21} \;\;\;D_{22} \;\;\; \cdots \;\;\;D_{2n} \hfill \\ \;\; \vdots \;\;\;\;\;\;\;\; \vdots \;\;\;\;\; \ddots \;\;\;\;\; \vdots \hfill \\ D_{n1} \;\;\;D_{n2} \;\;\; \cdots \;\;\;D_{nn} \hfill \\ \end{gathered} \right] \hfill \\ \end{gathered}$$

where $D_{{i_{j} }} = \left\{ {\left( {b_{1}^{{ij}} ,v_{1}^{{ij}} } \right),\left( {b_{2}^{{ij}} ,v_{2}^{{ij}} } \right), \ldots ,\left( {b_{n}^{{ij}} ,v_{n}^{{ij}} } \right)} \right\}$ $D_{{j_{i} }} = \left\{ {\left( {1 - b_{1}^{{ij}} ,v_{1}^{{ij}} } \right),\left( {1 - b_{2}^{{ij}} ,v_{2}^{{ij}} } \right), \ldots ,\left( {1 - b_{n}^{{ij}} ,v_{n}^{{ij}} } \right)} \right\}$ $b^{ij}_{k} \in [0,1]$; $D_{ii} = \left\{ {\left( {0.5,1.0} \right)} \right\}$; $\forall i,j,k \in \left\{ {1,2, \ldots ,n} \right\}$.$b^{ij}_{k}$ represent the importance degree of plan $i$ relative to plan $j$ as considered by the evaluator $k$, and $v^{ij}_{k}$ represents the support of the evaluator for the importance.

Since the D-number preference relation can avoid the influence of the difference of evaluators' experience and ambiguity on their results, the above cases can be expressed as:

$$R_{D1} = \left[ \begin{gathered} \left\{ {\left( {0.5,1.0} \right)} \right\} \left\{ {\left( {0.7,0.8} \right),\left( {0.6,0.2} \right)} \right\} \hfill \\ \left\{ {\left( {0.3,0.8} \right),\left( {0.4,0.2} \right)} \right\} \left\{ {\left( {0.5,1.0} \right)} \right\} \hfill \\ \end{gathered} \right]\;R_{D2} = \left[ \begin{gathered} \left\{ {\left( {0.5,1.0} \right)} \right\} \left\{ {\left( {0.8,0.6} \right)} \right\} \hfill \\ \left\{ {\left( {0.2,0.6} \right)} \right\} \left\{ {\left( {0.5,1.0} \right)} \right\} \hfill \\ \end{gathered} \right]$$

## D-AHP method

The analytic hierarchy process (AHP) makes a multi-criteria decision by clarifying the influencing factors of the analysis object and forming a hierarchical structure, and comparing the relative importance of the elements of the hierarchical structure. Incompleteness of evaluation information limits the use of AHP method. Therefore, the D-AHP method is obtained by deeply integrating the D-number preference relationship with the AHP method. That is, AHP method is used to construct the hierarchical structure. The index weights of all levels are solved by the D-number preference relation to evaluate the decision-making problem in complex and uncertain environment. The hierarchy structure of D-AHP includes target layer, criterion layer and scheme layer. The weight solution process is as follows:

Let the weight of criterion layer index $C_{j} \left( {j = 1,2, \ldots ,m} \right)$ relative to target layer be $w_{{c_{j} }}$, $w_{{c_{j} }} \ge 0$ and $\sum\limits_{j = 1}^{m} {w_{{c_{j} }} } = 1$; Let the weight of the evaluation object $A_{j} \left( {i = 1,2, \ldots ,n} \right)$ of the scheme layer relative to the indicator $C_{j}$ of the criterion layer be $w_{{A_{{i_{j} }} }}$, $w_{{A_{{i_{j} }} }} \ge 0$ and $\sum\limits_{{i = 1}}^{n} {w_{{A_{{i_{j} }} }} } = 1$. According to Eq. (9), the comprehensive weight of the evaluation object $A_{i}$ relative to the evaluation objective is solved:

9 $$\begin{gathered} w_{i} = \sum\limits_{j = 1}^{m} w_{{A_{{i_{j} }} }} w_{cj} ,w_{i} \ge 0\;{\text{and}}\;\sum\limits_{i = 1}^{n} {w_{i} } = 1 \hfill \\ \left( {i = 1,2, \ldots ,n,j = 1,2, \ldots ,m} \right) \hfill \\ \end{gathered}$$

## Determination of weight of emergency capability evaluation index

### Establishment of emergency capability evaluation system

The emergency response capability covers the whole process of pre-event prevention, incident response, in-process disposal and post-event management. Based on the research data of emergency management, an emergency response capability assessment system with 4 first-level indicators and 18 s-level indicators including prevention preparedness, monitoring and early warning, emergency response, accident handling and recovery is planned to be established. The hierarchy of each indicator is shown in Table 1.

**Table 1:** Emergency capability evaluation index and weight.

Target layer	Criterion layer	Comprehensive weight	scheme layer	Local weights	comprehensive weight
Emergency management capability indicator system	pre-event: Emergency Prevention and Preparedness (C_1_)	0.455	Organization Building and Evaluation (Z_1_)	0.133	0.061
			Risk Prevention (Z_2_)	0.288	0.131
			Allocation and expropriation of Emergency Resources (Z_3_)	0.093	0.042
			Publicity, Education and Training (Z_4_)	0.138	0.063
			Plan Preparation and Dynamic Management (Z_5_)	0.348	0.158
	Incidents: Surveillance and Early Warning (C_2_)	0.175	Event Monitoring and Early Warning (Z_6_)	0.435	0.076
			Information Report (Z_7_)	0.335	0.059
			Study on Early Warning Decision (Z_8_)	0.215	0.038
			Professional Team Management (Z_9_)	0.015	0.003
	Event: Emergency Response and Rescue (C_3_)	0.275	Advance disposal (Z_10_)	0.34	0.094
			Decision Support (Z_11_)	0.22	0.061
			Coordinated linkage (Z_12_)	0.26	0.072
			Government-public Partnership (Z_13_)	0.05	0.011
			Information Release and Public Opinion Guidance (Z_14_)	0.14	0.039
	Post: Recovery and Reconstruction (C_4_)	0.095	Restoration and Reconstruction (Z_15_)	0.1	0.010
			Aftercare Compensation (Z_16_)	0.45	0.043
			Survey Assessment (Z_17_)	0.3	0.029
			Emergency Responsibility Inspection (Z_18_)	0.15	0.014

### Weight calculation based on D-AHP method

1. Use the fusion formula (1) to convert the D-number matrix $R_{D}$ into the real number matrix $R_{C}$.
2. The probability matrix $R_{P}$ is constructed according to matrix $R_{C}$ to represent the preference probability between two elements.
  Denote the elements of matrix $R_{c}$ as $C_{ij}$, and denote the elements of matrix $R_{p}$ as: $P_{ij} = P_{r} (C_{i} \succ C_{j} ),\forall i,j \in \{ 1,2,...,n\}$.
  ① When $c_{ij} + c_{ji} = 1$, if $c_{ij} > 0.5$, then $\Pr (B_{i} \succ B_{j} ) = 1$;If $c_{ij} \le 0.5$, then $pr(B_{i} \succ B_{j} ) = 0$.
  ② When $c_{ij} + c_{ji} < 1$, if $c_{ij} \ge 0.5$, then $pr(B_{i} \succ B_{j} ) = 1$ and $pr(A_{j} \succ A_{i} ) = 0$;If $C_{ji} \ge 0.5$, then $pr(B_{i} \succ B_{j} ) = 1$ and $pr(B_{i} \succ B_{j} ) = 0$.
  ③ When $c_{ij} + c_{ji} < 1$, $c_{ij} < 0.5$ and $c_{ji} < 0.5$, unassigned preferences $c_{up} = 1 - (c_{ij} + c_{ji} )$. The probability that one indicator is superior to another is:
    10 $$pr(B_{i} \succ B_{j} ) = 1 - \frac{{(0.5 - c_{ij} )}}{{c_{up} }}$$
    11 $$pr(B_{j} \succ B_{i} ) = 1 - \frac{{(0.5 - c_{ji} )}}{{c_{up} }}$$
3. Calculate the sum of each row in matrix $R_{P}$, adjust the order of rows and columns in $R_{P}$ according to the size of each row sum, and obtain the triangulation matrix $R_{P}^{T}$.
4. According to the triangulation matrix $R_{P}^{T}$ triangulation of the real matrix $R_{C}$, the triangulated real matrix $R_{C}^{T}$ is obtained. Where, when the elements of $R_{C}^{T}$ meet: $R_{C}^{T} (i,j) + R_{C}^{T} (j,i) < 1$, $R_{C}^{T}$ needs to be further normalized to obtain the specification matrix $R_{{C^{\prime } }}^{T}$, and the formula is as follows:
  12 $$R_{C^{\prime}}^{T} (i,j) = R_{C}^{T} (i,j) + \frac{{1 - \left[ {R_{C}^{T} (i,j) + R_{C}^{T} (j,i)} \right]}}{2}$$
5. Calculate the weight of each index according to $R_{C}^{T}$ (or $R_{{C^{^{\prime}} }}^{T}$) matrix.

The weight is calculated by introducing parameter $\lambda$, which is related to the cognitive ability of experts. The specific value formula of $\lambda$ is as follows:

13 $$\lambda = \left\{ \begin{gathered} \left\lceil {\underline{\lambda } } \right\rceil ,\quad H{\text{ighly confidence in information}} \hfill \\ n,\quad \;\;\;\,M{\text{edium confidence in information}} \hfill \\ \frac{{n^{2} }}{2},\quad \;L{\text{ow confidence in information}} \hfill \\ \end{gathered} \right.$$

where $\underline{\lambda }$ represents the lowest bound of $\lambda$, $\left\lceil {\underline{\lambda } } \right\rceil = \min \left\{ {K \in Z\left| {K \ge \underline{\lambda } } \right.} \right\}$. For example, if $\underline{\lambda } = 1.67$, then $\lambda = \left\lceil {\underline{\lambda } } \right\rceil = 2$. $n$ represent the number of schemes.

### Weight calculation

According to Table 1, the weight of each index in the emergency capacity index system is calculated.

(1) Construct D matrix:
  $$\begin{gathered} \begin{array}{*{20}c} {\;\;\;\;\;\;\;\;\;\;\;\;\;\;\;\;\;\;\;\;\;\;\;\;\;\;\;\;\;\;\;\;C_{1} } & {\;\;\;\;\;\;\;\;\;\;\;\;\;\;\;\;\;\;\;\;C_{2} } & {\;\;\;\;\;\;\;\;\;\;\;\;\;\;\;\;\;\;\;C_{3} } & {\;\;\;\;\;\;\;\;\;\;\;\;\;\;\;\;\;\;\;\;\;\;\;\;\;\;\;C_{4} } \\ \end{array} \hfill \\ R_{D} = \begin{array}{*{20}c} {C_{1} } \\ {C_{2} } \\ {C_{3} } \\ {C_{4} } \\ \end{array} \;\left[ {\begin{array}{*{20}c} {\left\{ {\left( {0.5,1} \right)} \right\}} & {\left\{ {\left( {0.6,1} \right)} \right\}} & {\left\{ {\left( {0.6,0.2} \right),\left( {0.7,0.8} \right)} \right\}} & {\left\{ {\left( {0.8,0.6} \right),\left( {0.7,0.4} \right)} \right\}} \\ {\left\{ {\left( {0.4,1} \right)} \right\}} & {\left\{ {\left( {0.5,1} \right)} \right\}} & {\left\{ {\left( {0.4,1} \right)} \right\}} & {\left\{ {\left( {0.6,0.8} \right)} \right\}} \\ {\left\{ {\left( {0.4,0.2} \right),\left( {0.3,0.8} \right)} \right\}} & {\left\{ {\left( {0.6,1} \right)} \right\}} & {\left\{ {\left( {0.5,1} \right)} \right\}} & {\left\{ {\left( {0.8,1} \right)} \right\}} \\ {\left\{ {\left( {0.2,0.6} \right),\left( {0.3,0.4} \right)} \right\}} & {\left\{ {\left( {0.4,0.8} \right)} \right\}} & {\left\{ {\left( {0.2,1} \right)} \right\}} & {\left\{ {\left( {0.5,1} \right)} \right\}} \\ \end{array} } \right] \hfill \\ \end{gathered}$$
According to the fusion formula of D numbers, $R_{D}$ can be transformed into:
  $$\begin{gathered} \;\;\;\;\;\;\;\;\;\;\;\;\;\;\;\begin{array}{*{20}c} {\;\;\;\;C_{1} } & {\;\;\;C_{2} } & {\;\;C_{3} } & {\;\;\;C_{4} } \\ \end{array} \hfill \\ R_{C} = \begin{array}{*{20}c} {C_{1} } \\ {C_{2} } \\ {C_{3} } \\ {C_{4} } \\ \end{array} \;\left[ {\begin{array}{*{20}c} {0.5} & {0.6} & {0.68} & {0.76} \\ {0.4} & {0.5} & {0.4} & {0.48} \\ {0.32} & {0.6} & {0.5} & {0.8} \\ {0.24} & {0.32} & {0.2} & {0.5} \\ \end{array} } \right] \hfill \\ \end{gathered}$$
Construct probability matrix
  $$\begin{gathered} \begin{array}{*{20}c} {\;\;\;\;\;\;\;\;\;\;\;\;\;\;\;\;\;\;\;\;C_{1} } & {C_{2} } & {C_{3} } & {C_{4} } \\ \end{array} \hfill \\ R_{P} = \;\begin{array}{*{20}c} {C_{1} } \\ {C_{2} } \\ {C_{3} } \\ {C_{4} } \\ \end{array} \;\;\left[ {\begin{array}{*{20}c} 0 & 1 & 1 & 1 \\ 0 & 0 & 0 & {0.9} \\ 0 & 1 & 0 & 1 \\ 0 & {0.1} & 0 & 0 \\ \end{array} } \right] \hfill \\ \end{gathered}$$
Sort according to the sum size of each row in $R_{p}$:
  $$C_{1} \succ C_{3} \succ C_{2} \succ C_{4}$$
  $$\begin{gathered} \begin{array}{*{20}c} {\;\;\;\;\;\;\;\;\;\;\;\;\;\;\;\;\;\;\;C_{1} } & {C_{3} } & {C_{2} } & {C_{4} } \\ \end{array} \hfill \\ R_{T}^{P} = \begin{array}{*{20}c} {C_{1} } \\ {C_{3} } \\ {C_{2} } \\ {C_{4} } \\ \end{array} \;\;\left[ {\begin{array}{*{20}c} 0 & 1 & 1 & 1 \\ 0 & 0 & 1 & 1 \\ 0 & 0 & 0 & {0.9} \\ 0 & 0 & {0.1} & 0 \\ \end{array} } \right] \hfill \\ \end{gathered}$$
Convert $$R_{C}$$ into $$R_{C}^{T}$$
  $$\begin{gathered} \begin{array}{*{20}c} {\;\;\;\;\;\;\;\;\;\;\;\;\;\;\;\;\;\;\;\;C_{1} } & {\;\;\;C_{3} } & {\;\;C_{2} } & {\;\;\;C_{4} } \\ \end{array} \hfill \\ R_{C}^{T} = \begin{array}{*{20}c} {C_{1} } \\ {C_{3} } \\ {C_{2} } \\ {C_{4} } \\ \end{array} \;\;\left[ {\begin{array}{*{20}c} {0.5} & {0.68} & {0.6} & {0.76} \\ {0.32} & {0.5} & {0.6} & {0.8} \\ {0.4} & {0.4} & {0.5} & {0.48} \\ {0.24} & {0.2} & {0.32} & {0.5} \\ \end{array} } \right] \hfill \\ \end{gathered}$$
  Because in matrix $R_{C}^{T}$, $R_{C}^{T} \left( {2,4} \right) + R_{C}^{T} \left( {4,2} \right) = 0.8 < 1$. Therefore, $R_{C}^{T}$ is standardized:
  $$\begin{gathered} \begin{array}{*{20}c} {\;\;\;\;\;\;\;\;\;\;\;\;\;\;\;\;\;\;\;\;C_{1} } & {\;\;C_{3} } & {\;\;\;C_{2} } & {\;\;C_{4} } \\ \end{array} \hfill \\ R_{{C^{^{\prime}} }}^{T} = \begin{array}{*{20}c} {C_{1} } \\ {C_{3} } \\ {C_{2} } \\ {C_{4} } \\ \end{array} \;\left[ {\begin{array}{*{20}c} {0.5} & {0.68} & {0.6} & {0.76} \\ {0.32} & {0.5} & {0.6} & {0.8} \\ {0.4} & {0.4} & {0.5} & {0.58} \\ {0.24} & {0.2} & {0.42} & {0.5} \\ \end{array} } \right] \hfill \\ \end{gathered}$$
Calculate the index weight according to $R_{{^{C^{\prime}} }}^{T}$
  $$\left\{ \begin{gathered} \lambda \left( {w_{1} - w_{3} } \right) = 0.68 - 0.5 \hfill \\ \lambda \left( {w_{3} - w_{2} } \right) = 0.6 - 0.5 \hfill \\ \lambda \left( {w_{2} - w_{4} } \right) = 0.58 - 0.5 \hfill \\ w_{1} + w_{2} + w_{3} + w_{4} = 1 \hfill \\ \lambda > 0;w_{i} \ge 0,\forall i \in \left\{ {1,2,3,4} \right\} \hfill \\ \end{gathered} \right.$$

Solution:

$$\left\{ \begin{gathered} w_{1} = 1/4 + 0.205/\lambda \hfill \\ w_{2} = 1/4 - 0.075/\lambda \hfill \\ w_{3} = 1/4 + 0.025/\lambda \hfill \\ w_{4} = 1/4 - 0.155/\lambda \hfill \\ \lambda \in \left[ {0.62,\left. \infty \right)} \right. \hfill \\ \end{gathered} \right.$$

Then $$w_{1} \in \left[ {0.25,0.581} \right]$$, $$w_{2} \in \left[ {0.129,0.25} \right]$$, $$w_{3} \in \left[ {0,0.29} \right]$$, $$w_{4} \in \left[ {0,0.25} \right]$$.

The assessment expert group is composed of emergency management researchers and managers with rich management experience. The assessment information is highly reliable, so $\lambda = \left\lceil {\underline{\lambda } } \right\rceil = 1$. The index weight of criterion layer is solved as $w_{1} = 0.455$, $w_{2} = 0.175$, $w_{3} = 0.275$, $w_{4} = 0.095$. That is, the weight of emergency prevention and preparation is 0.455, the weight of monitoring and early warning is 0.175, the weight of emergency treatment and rescue in the event is 0.275, and the weight of post-recovery is 0.095.

Similarly, the weight of each element of the indicator layer can be obtained, and the results are shown in Table 1. Due to space limitation, no detailed calculation will be made.

### Result Analysis

For the first-level indicators, the importance of the 4 indicators from high to low is emergency prevention and preparedness (C_1_), emergency response and rescue (C_3_), surveillance and early warning (C_2_) and recovery and reconstruction (C_4_).

For the second-level indicators: ① pre-event prevention: Plan Preparation and Dynamic Management (Z_5_) $\succ$ Risk Prevention (Z_2_) $\succ$ Publicity, Education and Training (Z_4_) $\succ$ Organization Building and Evaluation (Z_1_) $\succ$ Allocation and expropriation of Emergency Resources (Z_3_); ② incident response: Event Monitoring and Early Warning (Z_6_) $\succ$ Information Report (Z_7_) $\succ$ Study on Early Warning Decision (Z_8_) $\succ$ Professional Team Management (Z_9_) ; ③ in-process disposal: Advance disposal (Z_10_) $\succ$ Coordinated linkage (Z_12_) $\succ$ Decision Support (Z_11_) $\succ$ Information Release and Public Opinion Guidance (Z_14_) $\succ$ Government-public Partnership (Z_13_); ④ post-event: Aftercare Compensation (Z_16_) $\succ$ Survey Assessment (Z_17_) $\succ$ Emergency Responsibility Inspection (Z_18_) $\succ$ Restoration and Reconstruction (Z_15_).

As emergency capacity building and upgrading requires as much human, material and advanced technical support as possible. Under the condition of being as objective as possible, the priority should be given to the construction of key elements according to the importance of quantified emergency capacity index, and then the construction of secondary elements should be supplemented and perfected. Then a dynamic, comprehensive, reasonable and efficient emergency system will be formed to meet the new needs of coal mine emergency management under the new situation.

## Evaluation of emergency capacity of coal mining enterprises

### Evaluation Process

According to the weight of each index determined by D-number theory and combined with Fuzzy Analytic Hierarchy Process (FAHP), the emergency capacity of a coal enterprise is evaluated.

1. Establish the evaluation factor set

The evaluation index system is constructed based on the factors that affect the emergency response capacity of coal mining enterprises, $U = \left\{ {U_{1} ,U_{2} , \ldots ,U_{n} } \right\}$. For each subset, $U_{i} = \left\{ {U_{i1} ,U_{i2} , \ldots ,U_{in} } \right\}$. The factor set for emergency response capacity evaluation is composed of 4 first-level indicators in Table 1, and the sub-factors are composed of 18 s-level indicators.

(2) Establish the weight set

Each factor has different degrees of influence on emergency response capability, and the degree of influence needs to be empowered for each factor. Determine the weight distribution of the factors at the next level to the factors at the previous level to form a weight set $W = \left\{ {W_{1} ,W_{2} , \ldots ,W_{n} } \right\}$, $\sum\limits_{i = 1}^{n} {W_{i} = 1}$ . According to the influence of each sub-factor to determine the weight of each sub-factor *W*_*i*_, $W_{i} = \left\{ {W_{i1} ,W_{i2} , \ldots ,W_{in} } \right\}$, *W*_ij_ ∈ [0,1]. *W*_ij_ represents the weight of *U*_ij_ in *U*_*i*_, and *n* represents the number of second-level indicators of *U*_*i*_.

(3) Establish the evaluation set

Establish the evaluation set: $V = \left\{ {V_{1} ,V_{2} , \ldots V_{n} } \right\}$. According to the characteristics of emergency response capacity, the comment set is divided into five levels: V = {*Excellent, Good, Average, Poor, Very poor*}.

(4) Determine the index membership degree

Index membership degree refers to the membership degree of each index to the evaluation set of coal enterprise emergency capacity. Suppose that x experts make quantitative evaluation on an index and y experts choose a comment level, then the membership degree is y/x.

(5) Determine the fuzzy relation matrix

According to the evaluation results of experts, the index membership can be obtained, that is, the fuzzy relation matrix $R = (r_{ij} )_{m \times n}$ can be generated. *r*_*ij*_ represents the membership of the *i* factor to the *j* element.

(6) Calculate the comprehensive evaluation matrix

In this paper, the weighted average type fuzzy operator is used to calculate the evaluation vector B of emergency capacity. The calculation is carried out layer by layer from the lowest level to the first level of the evaluation index system, and the calculation is as follows:

14 $$B = W \cdot R$$

(7) Quantitative assessment of the emergency capacity of coal enterprises

The 5 comments in the comment set are assigned, and the assignment matrix P is obtained, then $P = (P_{i} )_{5 \times 1}$.Then multiply the comprehensive evaluation matrix and the assignment matrix to obtain the quantitative evaluation value F of emergency response capability^37^.

15 $$F = B \cdot P$$

In order to make a more intuitive quantitative analysis of the evaluation results, the scores of the 5 comments are graded as shown in Table 2, and P is assigned as: P1 = 90, P2 = 80, P3 = 70, P4 = 60, P5 = 50.

**Table 2:** Comprehensive evaluation criteria for emergency response capacity.

Value (*P*)	[50,60)	[60,70)	[70,80)	[80,90)	[90,100)
Comment grade	Very poor	Poor	Average	Good	Excellent

### Example analysis

The experts’ scores on the subordination of the emergency response capacity of coal enterprises are shown in Table 3.

**Table 3:** Membership degree of second-level index.

Target layer	Criterion layer	scheme layer	Comment grade				
			Excellent	Good	Average	Poor	Very poor
Emergency management capability indicator system	pre-event: Emergency Prevention and Preparedness (C_1_)	Organization Building and Evaluation (Z_1_)	0.3	0.5	0.2	0	0
		Risk Prevention (Z_2_)	0.4	0.3	0.3	0	0
		Allocation and expropriation of Emergency Resources (Z_3_)	0.2	0.4	0.3	0.1	0
		Publicity, Education and Training (Z_4_)	0.3	0.4	0.3	0	0
		Plan Preparation and Dynamic Management (Z_5_)	0.3	0.4	0.2	0.1	0
	Incidents: Surveillance and Early Warning (C_2_)	Event Monitoring and Early Warning (Z_6_)	0.4	0.3	0.2	0.1	0
		Information Report (Z_7_)	0.5	0.4	0.1	0	0
		Study on Early Warning Decision (Z_8_)	0.3	0.4	0.2	0.1	0
		Professional Team Management (Z_9_)	0.3	0.3	0.2	0.1	0.1
	Event: Emergency Response and Rescue (C_3_)	Advance disposal (Z_10_)	0.4	0.5	0.1	0	0
		Decision Support (Z_11_)	0.4	0.3	0.3	0	0
		Coordinated linkage (Z_12_)	0.3	0.4	0.2	0.1	0
		Government-public Partnership (Z_13_)	0.3	0.4	0.1	0.1	0.1
		Information Release and Public Opinion Guidance (Z_14_)	0.2	0.4	0.3	0.1	0
	Post: Recovery and Reconstruction (C_4_)	Restoration and Reconstruction (Z_15_)	0.4	0.4	0.1	0.1	0
		Aftercare Compensation (Z_16_)	0.3	0.5	0.1	0.1	0
		Survey Assessment (Z_17_)	0.3	0.4	0.2	0.1	0
		Emergency Responsibility Inspection (Z_18_)	0.4	0.4	0.2	0	0

According to the membership degree and weight of each index, Eq. (14) can be used to calculate B_1_ = (0.320, 0.385, 0.252, 0.044, 0), B_2_ = (0.411, 0.355, 0.167, 0.067, 0.002), B_3_ = (0.345, 0.416, 0.199, 0.045, 0.005), B_4_ = (0.325, 0.445, 0.145, 0.085, 0). The membership matrix of the second-level index is:

$$R = \left[ \begin{gathered} B_{{1}} \, \hfill \\ B_{2} \hfill \\ B_{3} \hfill \\ B_{4} \hfill \\ \end{gathered} \right] = \left[ {\begin{array}{*{20}c} {{0}{\text{.320}}} & {{0}{\text{.385}}} & {{0}{\text{.252}}} & {{0}{\text{.044}}} & {0} \\ {{0}{\text{.411}}} & {{0}{\text{.355}}} & {{0}{\text{.167}}} & {{0}{\text{.067}}} & {{0}{\text{.002}}} \\ {{0}{\text{.345}}} & {{0}{\text{.416}}} & {{0}{\text{.199}}} & {{0}{\text{.045}}} & {{0}{\text{.005}}} \\ {{0}{\text{.325}}} & {{0}{\text{.445}}} & {{0}{\text{.145}}} & {{0}{\text{.085}}} & {0} \\ \end{array} } \right]$$

According to Eq. (14), the comprehensive evaluation vector can be obtained $B = W \cdot R$.

$$= \left( {0.{455},0.{175},0.{275},0.0{95}} \right)\left[ {\begin{array}{*{20}c} {{0}{\text{.320}}} & {{0}{\text{.385}}} & {{0}{\text{.252}}} & {{0}{\text{.044}}} & 0 \\ {{0}{\text{.411}}} & {{0}{\text{.355}}} & {{0}{\text{.167}}} & {{0}{\text{.067}}} & {{0}{\text{.002}}} \\ {{0}{\text{.345}}} & {{0}{\text{.416}}} & {{0}{\text{.199}}} & {{0}{\text{.045}}} & {{0}{\text{.005}}} \\ {{0}{\text{.325}}} & {{0}{\text{.445}}} & {{0}{\text{.145}}} & {{0}{\text{.085}}} & {0} \\ \end{array} } \right]$$

$$= (0.343,0.394,0.212,0.052,0.002)$$

According to Eq. (15), $$F = B \cdot P$$ = $$(0.343,0.394,0.212,0.052,0.002)$$$$\left[ \begin{gathered} {90 } \hfill \\ {80} \hfill \\ {70} \hfill \\ {60} \hfill \\ {50} \hfill \\ \end{gathered} \right]$$  = 80.45 can be obtained.

$$= {8}0.{45}.$$

Therefore, the emergency capacity of the enterprise is *Good*.

## Conclusion

(1) It is difficult to find a judgment matrix that meets the consistency when the evaluation is performed by AHP. It is difficult for experts to determine the membership degree of evaluation indicators, to evaluate uncertain information and incomplete information, and to avoid the interaction between evaluation indicators, which will affect the reliability of evaluation results. Therefore, the D-number theory is improved based on the D-S theory. Combining the theory of D-number with the analytic hierarchy process, the weights of 4 first-level indicators and 18 s-level indicators in the hierarchical structure model of emergency response capacity evaluation were calculated, and the weights and importance of each indicator were obtained. This effectively solves the evaluation under uncertain and unknown information, and avoids the interaction between evaluation indicators.

(2)According to the weight and importance calculation results of all levels of evaluation indicators, the importance of all level-1 and level-2 indicators was ranked respectively. Key indicators for emergency capacity building were identified. The fuzzy comprehensive evaluation method was used to construct the comprehensive evaluation matrix of emergency capability. Based on the results of evaluation experts and combined with the comprehensive evaluation and grading standard of emergency capability, the emergency capability of a coal enterprise was quantitatively evaluated. The final evaluation score was 80.45, and the evaluation result was "good", which was consistent with the actual situation of the enterprise's emergency response capacity. This provides a theoretical reference for the further construction and improvement of enterprise emergency capacity.

(3) According to the evaluation process of fuzzy analytic hierarchy process, the evaluation factor set, weight set and evaluation set of the secondary evaluation index are established. According to the expert evaluation results, the membership degree of the secondary index is determined, and then the comprehensive evaluation matrix is obtained. Combined with the importance of the secondary index and the assignment matrix, the paper makes a quantitative evaluation of the emergency capacity of coal enterprises. Finally, the overall evaluation score of the emergency capacity of a coal enterprise is 80.45 points, and the evaluation result is "good". The D-FAHP method can not only effectively identify the weak links in emergency management, but also meet the emergency decision-making needs of enterprises in an emergency state. It provides a new method for enterprise emergency capability assessment.

## Data availability

All data generated or analysed during this study are included in this published article.
